# Muscle metabolism and atrophy: let’s talk about sex

**DOI:** 10.1186/s13293-019-0257-3

**Published:** 2019-08-28

**Authors:** Megan E. Rosa-Caldwell, Nicholas P. Greene

**Affiliations:** Integrative Muscle Metabolism Laboratory, Exercise Science Research Center, Department of Human Health Performance and Recreation, University of Arkansas, Fayetteville, AR 72701 USA

**Keywords:** Muscle atrophy, Disuse, Cancer cachexia, Mitochondria, Hormones, Sex differences

## Abstract

Skeletal muscle health is a strong predictor of overall health and longevity. Pathologies affecting skeletal muscle such as cancer cachexia, intensive care unit treatment, muscular dystrophies, and others are associated with decreased quality of life and increased mortality. Recent research has begun to determine that these muscular pathologies appear to present and develop differently between males and females. However, to our knowledge, there has yet to be a comprehensive review on musculoskeletal differences between males and females and how these differences may contribute to sex differences in muscle pathologies. Herein, we present a review of the current literature on muscle phenotype and physiology between males and females and how these differences may contribute to differential responses to atrophic stimuli. In general, females appear to be more susceptible to disuse induced muscle wasting, yet protected from inflammation induced (such as cancer cachexia) muscle wasting compared to males. These differences may be due in part to differences in muscle protein turnover, satellite cell content and proliferation, hormonal interactions, and mitochondrial differences between males and females. However, more works specifically examining muscle pathologies in females are necessary to more fully understand the inherent sex-based differences in muscle pathologies between the sexes and how they may correspond to different clinical treatments.

## Background

Muscle makes up ~ 47–60% of lean body mass in men and women and is one of the greatest contributors to whole body energy expenditure [1]. Therefore, maintaining skeletal muscle health is critical to maintaining health and longevity throughout the lifetime. Various pathological conditions, such as prolonged periods of disuse, cancer cachexia, burn injuries, and others cause dramatic muscle atrophy, which in turn relates to a decrease in overall quality of life and increased mortality [2]. Specifically, disuse atrophy, a common occurrence with prolonged bed rest, casting, and space-flight, develops rapidly and significantly increases mortality and morbidity in these populations [2, 3]. For example, muscle loss occurs rapidly in intensive care unit (ICU) patients, and the degree of muscle loss is associated with increased treatment time and mortality [4–7].

Interestingly, these disuse-induced pathologies appear to discriminate between biological sexes, with females tending to have faster onset of muscle loss compared to males [8] which has been postulated to correspond to increased mortality in females [9]. However, until recently, investigations into disuse pathologies have primarily been conducted in only one sex [10–20] despite the influence of biological sex in many diseases [21–26]. However, the differences and similarities between males and females during atrophic pathologies is not currently codified. Therefore, the purpose of this review is to examine the current literature on various physiological processes between males and females potentially contributing to muscle health, including general muscle phenotype, response to catabolic and anabolic stimuli, hormonal contributions to muscle health, as well as mitochondrial profiles.

### Muscle phenotypes between males and females

**Fig. 1:**
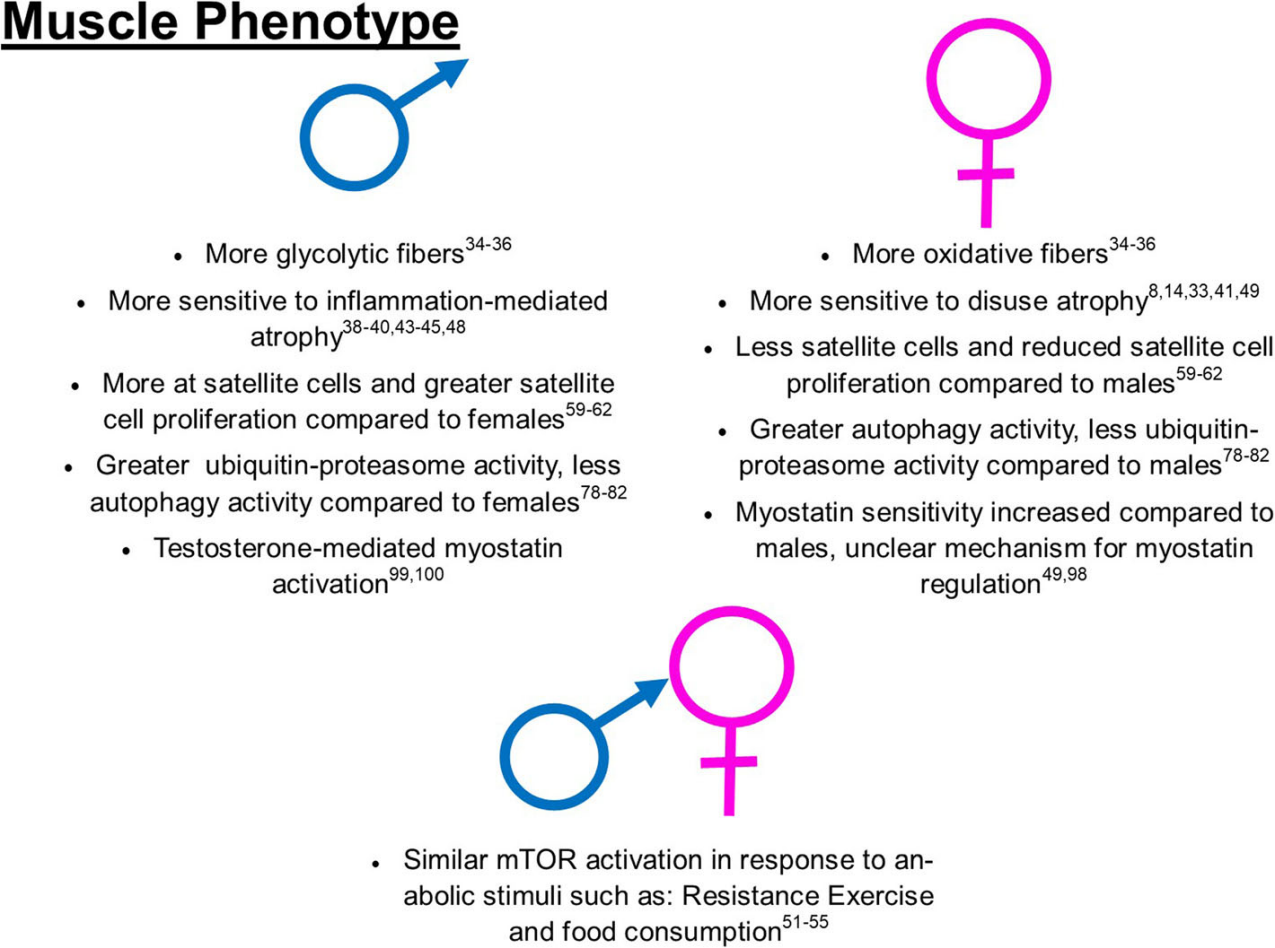
Summary of current literature of sex differences and similarities of muscle phenotype in males and females

The maintenance of muscle size relies on a delicate balance between protein synthesis and degradation, whereby an increased protein synthesis:degradation ratio results in muscle hypertrophy and decreased synthesis:degradation ratio results in muscle atrophy. Many diseases are associated with reduced health outcomes with muscle loss [27, 28]. Recent works have established that mechanisms contributing to these muscle pathologies are different depending on the disease, making effective treatment options difficult. For example, cancer is also known to cause marked muscle and body fat loss, with body weight losses being strongly associated with mortality. However, this particular form of atrophy appears to be related to the inflammatory action of the tumor-host interactions [29–32], whereas disuse atrophy does not typically display this classical inflamed phenotype [33]. Until recently, it was thought that processes contributing to muscle loss were not sex specific. However, recent works have begun to establish differences between males and females on responsiveness to atrophic and hypertrophic stimuli. In this section, we will specifically focus on sex differences in overall muscle phenotype and how these differences may contribute to differential sensitivity to catabolic and anabolic factors as well as highlight areas for further research (Fig. 1).

#### Fiber type differences between males and females and susceptibility for disuse atrophy

Data have begun to demonstrate muscle composition differences between males and females. Females tend to rely more on oxidative metabolism compared to males [34]; correspondingly, females also have greater relative content of type I muscle fibers compared to males within the same muscle [35, 36]. Works have previously established that different muscle pathologies differently affect different muscle fibers [37], with cancer cachexia more strongly influencing glycolytic fibers compared to oxidative [38–40] and disuse atrophy preferentially selecting for oxidative fibers [14, 33, 41]. Although it is not completely understood why disuse atrophy more radically impacts oxidative fibers, the differential content of fibers between males and females may be an important consideration for future research investigating sex as a biological variable with muscle atrophies.

#### Disuse muscle loss between males and females

While muscle loss occurs in both males and females across a variety of pathologies, different pathologies appear to differentially affect males and females [8, 9, 42–46]. For example, during aging-induced atrophy, females experience a greater shift toward smaller fibers compared to males [47]. However, during inflammation-based muscle pathologies, such as cancer cachexia, males tend to have greater muscle losses and subsequent side effects compared to females [43–45, 48]. Overall, these works suggest that males and females exhibit differing muscle atrophy responses depending on the precise stimuli. Specific to disuse atrophy, female mice have been shown to exhibit a greater percent loss of soleus mass compared to males during hindlimb unloading [49], suggesting females may be particularly susceptible to disuse atrophy compared to males. However, it is not currently clear if this is due to a higher percentage of disuse-susceptible type I fibers in females or innate differences in muscle physiology. Clinically, recent works suggest that females are more likely to succumb to intensive care unit (ICU)-associated muscle weakness [8], potentially leading to more ICU-associated deaths [9]. Taken together, these data clearly suggest different responses to atrophic stimuli between males and females and highlight the need for further research investigating mechanisms for muscle wasting across a variety of pathologies.

#### Sex difference in anabolic and catabolic factors

As the maintenance of muscle mass depends on the balance of anabolic and catabolic factors, here we will briefly describe known differences between males and females on mediators of protein synthesis and degradation. Briefly, one of the major mediators of protein synthesis is the mammalian target of rapamycin (mTOR). Activation of mTOR by anabolic stimuli (resistance exercise, insulin, amino acids, etc.) results in phosphorylation of 4EBP-1 and S6K1 proteins, allowing for mRNA translation and subsequent protein synthesis [50]. In general, most studies have found that males and females have similar mTOR activation and subsequent protein synthesis with anabolic stimuli such as food consumption or resistance exercise [51–55]. However, there still remains some controversy on sex differences with regards to anabolic processes. For example, some literature has suggested increased protein synthesis measured by fractional myofibrillar synthesis rates in females compared to males with whey ingestion [56], whereas others have found males to have greater increases in muscle protein synthesis compared to females after weeks of sprint interval training [57]. Regardless, the current literature tends to favor no sex-mediated differences on mTOR activation and subsequent protein synthesis [51–55].

Conversely, recent evidence suggests potential sex differences in satellite cell activation and proliferation. Briefly, satellite cells are myogenic stem cells, activated during regenerative stimuli, such as resistance exercise or muscle damage [58]. Once activated, satellite cells proliferate to form new myonuclei and facilitate muscle hypertrophy or regeneration, depending on the specific stimuli [58]. A more through overview of satellite cell activation and proliferation has been reviewed elsewhere [58]. Male satellite cells appear to have more mRNA related to differentiation and hypertrophy such as myogenin and MyoD compared to females [59] and appear to also have greater proliferative capacity compared to females [60, 61]. For example, it was recently found that young male mice tend to have greater numbers of satellite cells compared to female mice [61], satellite cells derived from male poultry also have greater proliferation in vitro [60]. These differences may in part be due to differences in testosterone-mediated satellite cell proliferation [62]. For example, in vitro culture of satellite cells treated with serum from castrated males demonstrate reduced proliferation compared to serum from uncastrated males [63]. In vivo loss of testosterone in various mammalian species results in reduced satellite cell content and reduced muscle size [64, 65]. Furthermore, adding supplemental testosterone in males mitigates these aberrations and can result in greater overall satellite cell content compared to control animals [64–66]. This hypothesis of testosterone-mediated satellite cell proliferation is further corroborated by research finding testosterone treatment in female mice produced an increase in the number of myonuclei [67]. Taken together, the aggregate of these studies strongly suggest that testosterone mediates greater satellite cell content in males, possibly allowing for greater muscle size. However, we should note that there has been recent controversy on the necessity of satellite cells for maintenance of muscle mass and hypertrophy [68–70]. Such controversies are beyond the scope of this review article; for further information, the reader is directed to excellent reviews on this topic [58, 71]. Overall based on the current literature, females and males do not appear to have clinically meaningful differences in mTOR activation and subsequent protein synthesis in response to anabolic stimuli; however, males do appear to have greater satellite cell content and proliferative capacity compared to females.

Catabolic factors and signaling mechanisms also play a large role in the overall muscle size. Broadly speaking, the two primary protein degradative pathways include ubiquitin-proteasome and autophagy pathways [72]. Briefly, the ubiquitin-proteasome system involves tagging old or misfolded proteins with small ubiquitin proteins; these ubiquitin-tagged proteins are then transferred to the proteome for degradation [73]. This system is regulated by various ligases (referred to as E1, E2, and E3 ligases) that facilitate activation of the ubiquitin, transfer of the ubiquitin to the target protein, and finally the attachment of ubiquitin to the target protein respectively [73].The ubiquitin-tagged protein is then shuttled to the proteasome for degradation and recycling of amino acids [73]. MuRF1 and Atrogin1 are important E3 ligases within the ubiquitin-proteasome pathway and are activated across a variety of catabolic muscle pathologies [73–75]. Similarly, the autophagic degradative pathway serves to remove damaged or old proteins [76]. Autophagy-related genes (Atgs) are the primary regulators of this pathway. Broadly speaking, catabolic stimuli signal to ULK1 protein to initiate autophagosomal formation, which is initiated by the Beclin (Atg6) protein [77]. Autophagosomal formation is facilitated by the activation of the LC3I to LC3II conversation induced by Atg4 [77]. Concurrently, the p62 (SQSTM1) cargo protein brings the proteins tagged for autophagosomal degradation to the autophagosome. Finally, the autophagosome binds with a lysosome for lysosomal degradation of the protein [77]. A more through overview of these process has been reviewed elsewhere [73, 77].

Recent works have begun to tease out the nuances in possible differences of these pathways between males and females. In general, it appears that both of these processes are at least partially mediated by biological sex, as prior works have found mRNAs related to these processes are differentially expressed between males and females [78]. Specifically females overall tend to have lower basal ubiquitin-proteasome activity compared to males, which is thought to be partially mediated by estrogen signaling [79, 80]. This lowered ubiquitin-proteasome activity appears to result in differential FOXO3a-Ubiquitin signaling during disuse atrophy, with females showing less FOXO3a content compared to males, yet greater ubiquitinated protein content [49]. Potential reasons or mechanism for this differential signaling during disuse have yet to be elucidated. While females tend to have less ubiquitin-proteasome activity compared to males, females tend to have greater autophagy-related protein degradation compared to males [81, 82]. Current literature suggests greater protein content of autophagy regulators of autophagy initiation and resolution compared to males [81, 82], overall suggesting that males and females may preferentially favor different protein catabolic pathways.

Specific to disuse atrophy, both males and females have potent inductions of degradative pathways with disuse [83, 84] [10, 12, 85–93]. However, these responses may be partially mediated by biological sex. For example, male rats and mice undergoing hindlimb unloading have increased Atrogin1 and MuRF1 induction in the early stages of disuse (~ 2–10-fold depending on length of unloading and tissue) [10, 12, 85–90]. Comparatively, females potentially have an augmented response compared to males, with some studies reporting as much as 20–40-fold increases in MuRF1 and Atrogin1 respectively [83, 84], though others have noted only 2–4-fold greater Atrogin1 and MuRF1 mRNA in hindlimb casted females [91]. Taken together, these studies may suggest similar degradative pathways in males and females during disuse atrophy; however, in females, degradative pathways such as ubiquitin-proteasome may be relatively greater compared to males. However, to our knowledge, this hypothesis has not been directly tested and may warrant further study. Similarly, autophagy is also known to be activated during disuse atrophies in both human and animal models [91, 92, 94]. However, potential sex differences in autophagy induction during disuse atrophy have not been directly evaluated between sexes.

Finally, males and females may differ in regulators of protein catabolism, specifically myostatin sensitivity. Myostatin, a protein within the TGF-β family, can greatly impact muscle size, with increased myostatin activity contributing to reduced muscle size in multiple models of muscle catabolism [95–97]. While myostatin will affect both sexes, females appear to be more responsive to myostatin withdrawal, with inhibition of myostatin causing greater muscle hypertrophy in females compare to males [98]. However, myostatin action in both sexes still remains perplexing as recent works have found disuse atrophy to increase myostatin content in females but not males [49]. This may be partially due to myostatin-androgen receptor reciprocity. Prior works have found myostatin translation and secretion moderated by androgen receptor translocation to the nucleus with androgen binding [99, 100]. Speculatively, this interaction between androgen receptor activity and myostatin synthesis may serve to counter balance androgen hypertrophic action. This hypothesis would align with noted differences in myostatin content between males and females during disuse atrophy; however, more works are needed to fully elucidate these complicated interactions.

Overall, the current literature appears to suggest multiple differences in muscle physiology between males and females, as well as a few similarities between the biological sexes. Males and females appear to have similar inductions of protein synthetic pathways with anabolic stimuli and both have large inductions of catabolic pathways with disuses; however, the relative strength of these inductions may warrant further research. Females tend to have more oxidative type I fibers compared to males [34–36], which may predispose females to greater muscle losses with disuse atrophy. Females tend to have less satellite cells [60, 61], which may be partially mediated by testosterone-activation of ARs on the satellite cells [64–66]. Females also tend to have less basal ubiquitin-proteasome activity and greater autophagy activity compared to males [79–82]. Finally, females appear to have differential regulation of myostatin activity and subsequent alterations to muscle physiology [49, 98]. Taken together, the aggregate of these data suggest differential interactions between muscle pathologies and biological sex, strongly suggesting the need for research investigating treatment interventions that optimize specific cellular mechanisms that differ between males and females in order to optimize therapies for musculoskeletal pathologies based on biological sex.

### Hormonal interactions

**Fig. 2:**
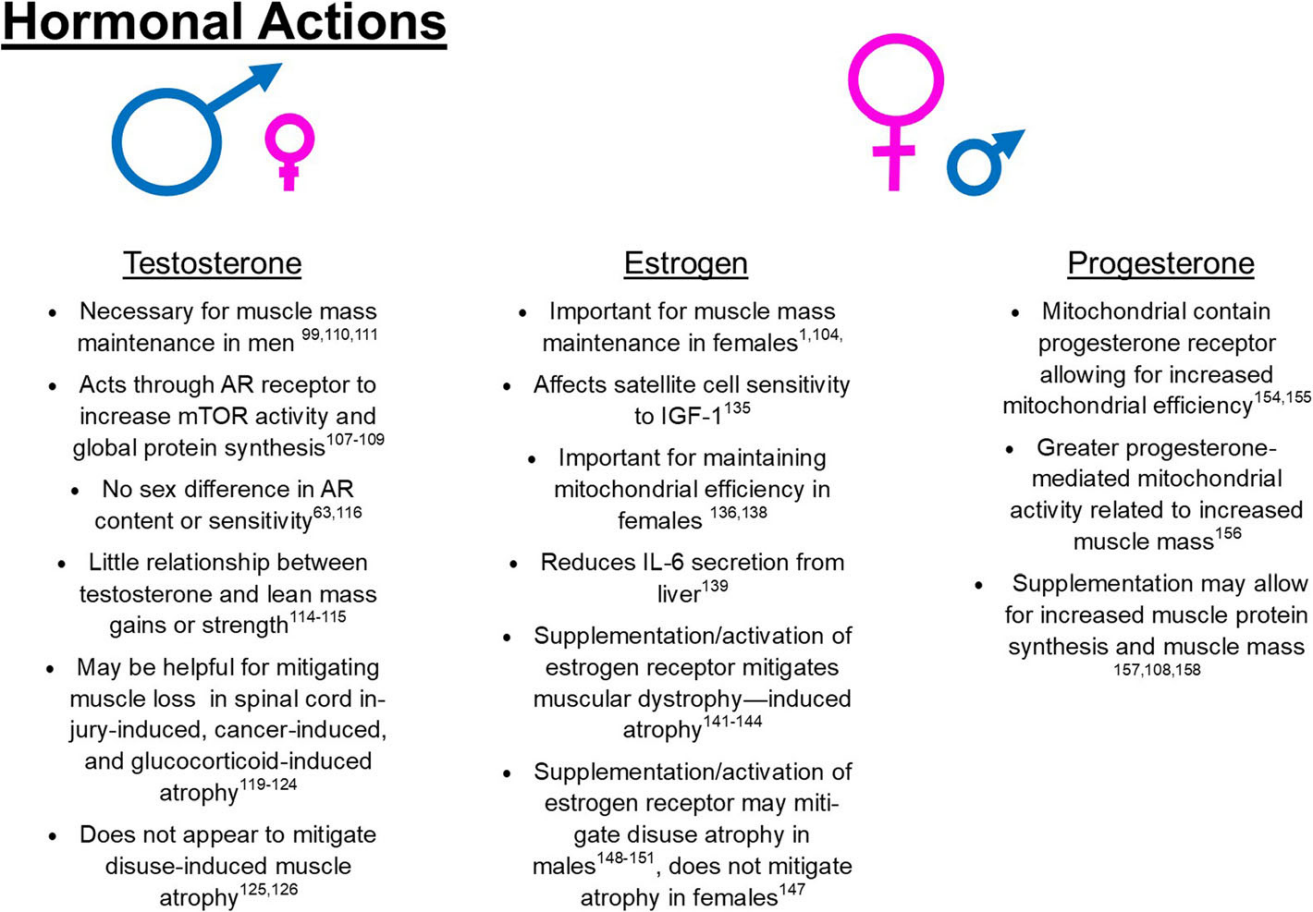
Summary of the current literature of classical sex hormones and their influence on muscle size.

One significant difference between males and females that likely contributes to dimorphic responses between sexes during muscle pathologies is differing circulating hormones. The process of synthesizing these hormones begins at the gonadotropic cells in the anterior pituitary gland of the brain. Gonadotropes facilitate signaling to sex organs for the synthesis of sex hormones. Different genetic expression within the gonadotropes at the anterior pituitary gland vary in mammals depending on age and menstrual/estrous cycling status. Among females, greater activation of gonadotrope signaling to sex organs is present during proestrus (corresponding to increased estrogen and progesterone synthesis) compared to diestrus [101]. In general, sex hormones exert beneficial effects on muscle mass maintenance and function [102–104]; however, their specific impacts on skeletal muscle may have specificity to the sex of the organism. While it is unlikely that hormones solely mediate differences in muscle health, it is likely that hormonal interactions have at least some influence on muscle quality and health. For the purpose of this review, we will focus primarily on the three primary sex-related hormones: testosterone, estrogen, and progesterone (Fig. 2).

#### Testosterone as a mediator of muscle growth

Testosterone is currently one of the most popular supplements in the USA, with sales of testosterone supplements increasing 500% between 1993 and 2000 [105], and continuing to grow. Men tend to have greater testosterone than women, with a normative range of ~ 82–257 ng/dL for young men and 0.8–10 ng/dL for young women [106]. Testosterone is an androgenic hormone that works through the action of androgen receptors (AR) on various tissues to facilitate protein anabolism. Specifically, part of this anabolic response involves AR translocation to the nucleus to act as a transcription factor to increase myotube and muscle protein synthesis [107, 108]. Previous work has demonstrated that testosterone can work specifically through ARs to contribute to increased protein synthesis [107, 108]. In human primary myotubes from men, recent work has clarified the pathways for testosterone action [107]. Specifically, it has been demonstrated that testosterone action can activate mTOR through androgen-mediated action of androgen receptor to activate PI3k/Akt signaling [107]. AR receptor content varies based on the type of tissue, and in muscle by the fiber type [109]. Interestingly, AR activation seems to favor a shift toward slow twitch fibers, with AR-knockout mice demonstrating a shift toward type II fibers [99]. In animal models, removing testosterone in male mice by castration induces a noted decrease in muscle size [99, 110, 111]. Whereas, when testosterone is given to those with low levels of testosterone, such as men with hypogonadism or older male mice, there appears to be increased muscle mass [110, 112]. In females, testosterone also appears to have anabolic effects, for example postmenopausal women given an acute treatment with testosterone exhibit increased fractional protein synthesis rates [113]. However, that same study found greater FSR in older females compared to young [113] as such, it is unclear if increasing protein synthesis rates alone necessitates a phenotypic change in the muscle in this population.

Whereas AR content appears necessary for appropriate muscle fiber size, it does not appear sufficient to induce muscle hypertrophy; for example, resistance exercise training does not appear to impact the number of AR [114]. Additionally, free testosterone in the blood has little relationship to changes in lean body mass or strength [114, 115]. Finally, there does not appear to be sex differences in the number of ARs or sensitivity to anabolic stimuli [63, 116], suggesting that sex differences in androgenic activity are likely due to higher concentrations of androgens in the blood.

During multiple muscle pathologies, hypogonadism and associated testosterone concentrations are known to be lowered and hypothesized to partially contribute to muscle loss [117–121]. For example, men with spinal cord injury demonstrate generally lower testosterone levels compared to non-injured controls [122]. However, testosterone supplementation in these populations has shown promise for mitigating this muscle loss in men [119, 120]. Hypogonadism also commonly accompanies cancer cachexia [117, 118], and recent works have begun to evaluate the sufficiency of drugs with androgen-like binding and testosterone itself as pharmacological agents for the treatment of cancer cachexia in men [123, 124]. Finally, testosterone supplementation appears to limit glucocorticoid-induced muscle atrophies in male mice [121]. However, despite these promising findings in other muscle pathologies, testosterone status does not appear to influence muscle mass recovery from hindlimb unloading, in that androgen supplementation in these populations does not mitigate muscle losses [125, 126]. Therefore, while the presence of testosterone appears to be a necessary component for muscle mass and quality in males, the precise relationships between testosterone status and muscle quality appear dependent on the specific muscle pathology. Overall, it does not appear that testosterone treatment is necessarily effective for mitigating disuse-induced muscle loss in males. However, mechanisms for these discrepancies between different muscle pathologies remain under investigated and warrant further research.

#### Estrogen as a mediator of muscle growth

Estrogen (also sometimes referred to as estradiol) is primarily associated as a female sex hormone whose concentration varies throughout the course of the menstrual (human) or estrous (murine) cycle. Specifically, women tend to have increased estradiol and progesterone during the luteal phase of menstrual cycle (300 pg/ml estradiol/day and 10 ng/ml progesterone/day during the luteal phase vs. 50 pg/ml estradiol/day and 1 ng/ml progesterone/day during the follicular phase, respectively) [127]. In female mice, estradiol and progesterone appear to peak at 40–60 pg/ml and 25–30 ng/ml respectively during proestrus and 7–10 pg/ml and 5 ng/ml during diestrus [128–132]. Each of these phases affects energy utilization and bioenergetics [133]. Estrogen overall appears to exert a hypertrophic effect [134], which is at least partially mediated by increased sensitivity of IGF-1 in satellite cells [135]. Estrogen also affects mitochondrial health [136, 137], whereby estrogen removal in ovariectomized rats decreases mitochondrial O_2_ consumption and efficiency [136, 138], overall suggesting that estrogen plays an important role for cellular and likely muscular health in females.

While the specific role of estrogen on muscle size and quality is still controversial, in general, deficiency of estrogen is likely detrimental to muscle function in both males and females [1]. Generally, ovariectomized young mice and postmenopausal women have smaller muscles compared to functional uterus controls [1], suggesting that estrogen plays a role in muscle size, at least in females. Estrogen also may be important for protection from atrophic stimuli. For example, during inflammation-induced atrophy, estrogen diminishes IL-6 secretion from the liver and appears to blunt atrophy [139]. This finding strongly suggests protective effects of estrogen and may partially account for differences seen between males and females during pathologies such as cancer cachexia, whereby cycling females do not experience cachexia compared to acyclic females who do undergo cachexia [140]. Estrogen-based treatments have begun to be investigated for various muscular dystrophies [141–144], specifically the use of tamoxifen, a drug developed for estrogen-dependent breast cancer. Finally, loss of estrogen receptors delays muscle regeneration and differentiation after injury [145]. Overall, the aggregate of the literature appears to suggest that estrogen is a necessary component of muscle size, with reduction or elimination of estrogen causing reduced muscle size and regenerative capacity. However, it should be noted that one study suggested activation of estrogen receptors in males to cause muscle atrophy through increased ubiquitin-specific peptidase 19 content and potential augmented activation of the ubiquitin-protease system [80], and recent work suggests that estrogen treatment in males can alter MHC gene expression as measured through microarray analysis in cardiac tissue [146]. Therefore, more research may be necessary to understand the intricacies of estrogen’s precise effects on muscle in both males and females.

Specific to disuse muscle atrophy, estrogen treatment and/or supplementation has shown mixed efficacy on protections against disuse-induced muscle pathologies. For example, ovariectomized mice showed delayed recovery from disuse-induced atrophy, and greater muscle loss (measured by cross sectional area) compared to ovariectomized mice with estrogen replacement [104], suggesting that estrogen may be a necessary component to mitigate the intensity of muscle atrophy. However, other work shows estrogen treatment in female mice to have little effect on muscular deteriorations with disuse [147], suggesting that estrogen treatment in females is not sufficient to counteract disuse atrophy. Contrastingly, estrogen-based treatments (including both estrogen and estrogen-receptor agonists) have shown some protections against disuse atrophy in male mice and rats [148–151]. Further demonstrating the necessity to investigate potential treatments against muscle pathologies in both male and female models.

Overall, the current literature appears to suggest that estrogen is a major mediator of muscle quality, with loss of estrogen associated with decreases in muscle quality. Supplementation of estrogen may increase muscle size in animals; however, more research using both male and female models are necessary to understand the full influence of estrogen on muscle quality and size.

#### Progesterone and muscle health

Similar to estrogen, progesterone also is associated as a female sex hormone, with massive increases in progesterone concentrations during the luteal phase of the menstrual cycle. However, compared to estrogen, progesterone has not been studied as extensively in relation to muscle quality.

One of the early investigations of progesterone found progesterone treatment on myotubes increased hydrogen peroxide (ROS) emission from the mitochondria [152], which the authors interpreted as pathological. However, based on this study, it is not clear whether this emission is necessarily pathological, as small amounts of ROS production may benefit mitochondrial and muscle health as seen in exercise studies where blunting ROS response blunts exercise adaptations [153]. Recently, is has been suggested that mitochondria contain a progesterone receptor that can directly mediate and increase mitochondrial respiration and efficiency [154, 155]. This increased respiration in the muscle may allow for the increased ATP production necessary to develop and maintain muscle mass. However, to our knowledge, this relationship has not been directly tested in mammalian species.

With specific regard to muscle hypertrophy, high feed efficiency broiler chickens have been found to have higher mitochondrial efficiency which appears to be mediated by progesterone action [156]. This work adds to the growing body of literature that mitochondrial health is a major mediator of muscle quality and this quality is at least partially mediated by progesterone action. Additionally, in cattle, feed efficiency can be increased ~ 20% by supplementation with progesterone [157], and progesterone treatment in mice and humans can stimulate cardiac and skeletal muscle protein synthesis [108, 158]. However, to our knowledge, no work has directly investigated the role of progesterone on disuse or other muscle atrophies. Based on works demonstrating progesterone’s hypertrophic capacity, progesterone treatment may be an additional treatment target for pharmacological interventions for the prevention of muscle atrophies. However, more works are needed to thoroughly understand progesterone’s mechanism of action for regulation of muscle quality.

#### Estrous cycle in relation to muscle pathologies

Recent literature is beginning to examine the influence of estrous/menstrual cycle on various pathologies and treatments in females. Currently, the aggregation of literature suggests that estrous cycle can influence some pathologies. For example, female mice are more sensitive to the effects of antidepressants during proestrus [159, 160] and other pathologies such as autoimmune diseases are thought to be mediated by estrogen and the estrous/menstrual cycle [161, 162], suggesting that hormonal status may influence some disease pathologies. Specific to muscle pathologies, recent work has strongly suggested that estrous cycle may influence development of cancer cachexia in female mice, whereby it is hypothesized that cancer cachexia may influence estrous cycling and result in muscle wasting [140, 163]. For example, in female Apc^min/+^ mice, 100% of acyclic females become cachectic as opposed to essentially no cycling females becoming cachetic [140]. This acyclicity appears to occur in ~ 38% of female Apc^min/+^ mice [140]; however, mechanisms for this cessation are not yet fully understood. These works suggest that the presence or absence of the estrous cycle influences muscle maintenance in cancer cachexia; however, these differences develop over the course of weeks. With the relatively short time frame of many disuse studies (~ 3–14 days) [10, 11, 92, 164], and the accelerated development of atrophy therein, it is unlikely that the presence or absence of cycling dramatically influences the progression of disuse atrophy. However, this hypothesis has not been directly investigated, therefore more research directly investigating the influence of estrous cycle during disuse atrophy is likely warranted.

Taken together, the aggregate of the current literature suggests that hormones can significantly influence overall muscle health and size. With loss of testosterone or estrogen dramatically influencing muscle size in males and females respectively [1, 104, 117–122, 134, 145]. However, the potential for supplementation or pharmacological activation of these specific receptors has mixed results depending on the sex of the model organism and muscle pathology [125, 126] [119–121, 123, 124, 141–144]. These data imply the necessity of research using both male and female organisms to more fully elucidate the influences of these hormones on muscle pathologies and potential treatment efficacy. Finally, progesterone, the traditionally classified female hormone has recently emerged as a potential regulator of mitochondrial and muscular health [154–156]. These recent findings warrant further research on the potential mechanisms of progesterone during muscle pathologies and possible therapeutic applications of progesterone during various muscle pathologies.

### Mitochondrial differences between males and females

**Fig. 3:**
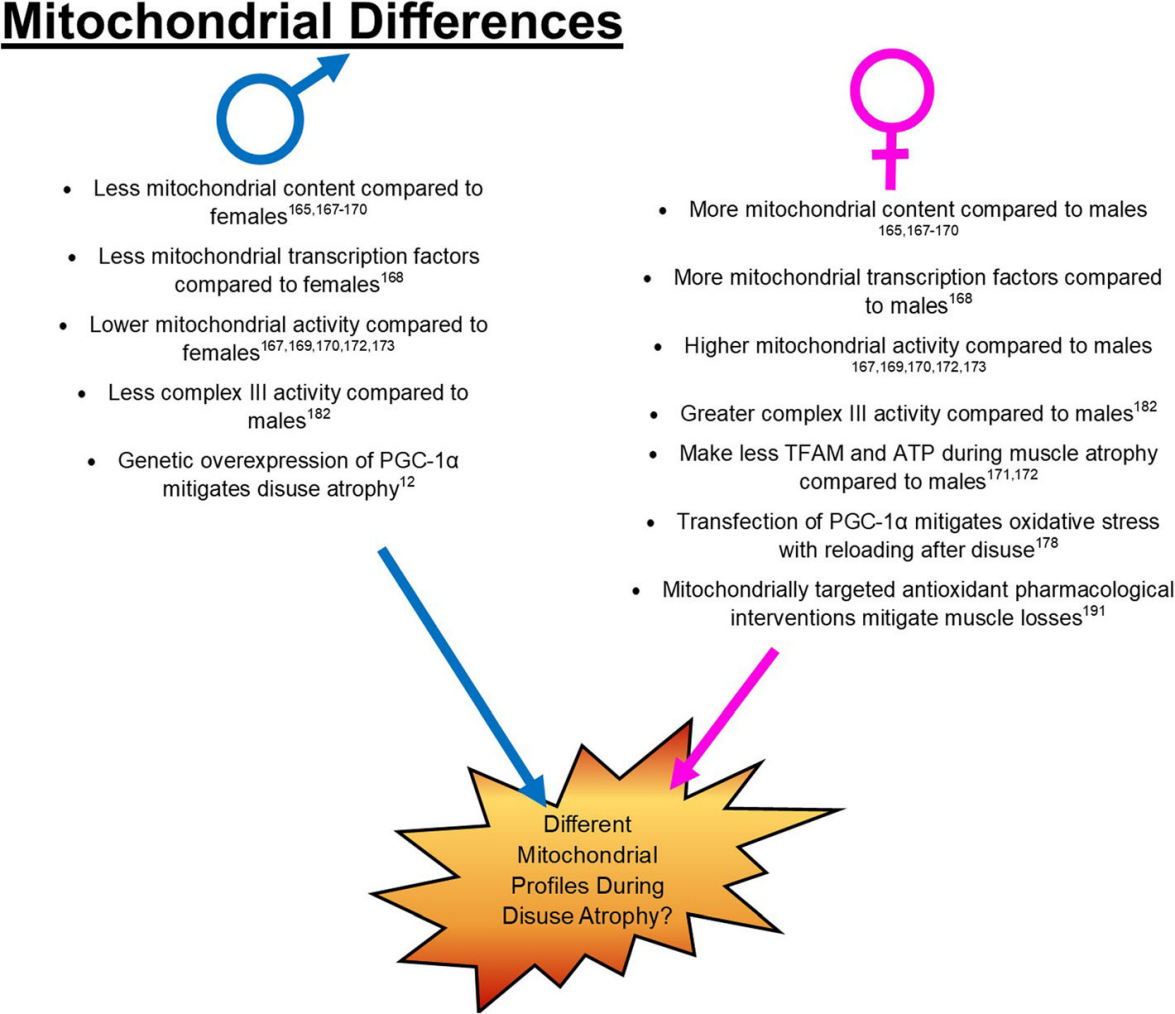
Summary of current data on mitochondrial differences between males and females as well as mitochondrial interventions for disuse atrophy

Mitochondrial quality has been a recently proposed mediator of muscle size and function [108]. Females in general tend to preferentially oxidize fat as the primary energy substrate [34, 165, 166], aligning with a greater relative content of type I fibers [35, 36]. Therefore, suggesting females’ higher reliance on mitochondrial oxidative phosphorylation for ATP synthesis. To date, multiple studies have found mitochondrial function, morphology, and content to differ between males and females. Indeed, animal and human studies across multiple tissues have found higher mitochondrial content per gram of tissue and greater transcription factors associated with mitochondrial biogenesis in females compared to males [165, 167–170]. Female gastrocnemius muscles have greater mitochondrial content and mtDNA per gram of tissue compared to males as well as more ATP synthase, TFAM protein, and mitochondrial complexes [168]. These differences appear to correspond to greater mitochondrial activities [167]. Additionally, in other tissues, such as the liver, females have greater mitochondrial content, and respiratory capacity [169, 170]. Taken together, these data demonstrate that some aspects of mitochondrial quality appear inherently different between males and females, which may contribute to differential progressions of mitochondrially related pathologies including muscle atrophies (Fig. 3).

#### Sex differences in mitochondrial aberrations during catabolic stimuli

Recent data suggests that mitochondrial alterations during catabolic stimuli appear to discriminate between males and females. During arthritis-induced disuse, females experience a large decline in subsarcolemmal mitochondrial density compared to males [35, 46]. Additionally, females have greater reductions in the mitochondrial translation protein TFAM compared to males with aging-associated muscle wasting [171] and female mitochondria tend to make less ATP compared to males during energetic stress such as glucose deprivation in vitro [172]. Overall demonstrating that females and males have different mitochondrial responses to cellular stress, emphasizing the need to further understand how female muscle differs from male, both during basal and stressed conditions. Recent research examining primary cells from males and females have demonstrated sexual dimorphisms on measures of mitochondrial function [172, 173], clearly demonstrating that sex differences are not solely hormone mediated and need to be further investigated using in vivo and in vitro methods. Overall, while it is generally well known that females have differing mitochondrial profiles compared to males, both at baseline and during differing pathologies, it is not directly clear how these differences may influence overall phenotypic and clinical outcomes.

#### Mitochondrial aberrations during disuse atrophy

Research throughout the past 25 years has demonstrated that disuse atrophy increases reactive oxygen species (ROS) and peroxide species production [13, 15–19, 174]. Because mitochondria are the primary generators of ROS, efforts to increase mitochondrial quality and function are being investigated as a therapeutic modality for disuse atrophy. For example, multiple studies have investigated the therapeutic potential of antioxidant treatments such as vitamin E supplementation. However, the results of these studies have been mixed with some data showing vitamin E supplementation to be protective [20, 175], whereas others have not seen any protective effects of supplementation [12, 13]. Additionally, because alterations in PGC-1α, mitochondrial quality, and oxidative phosphorylation have been noted as substantial contributors to disuse muscle wasting [14, 91, 176], efforts to improve these markers have been investigated as possible therapeutic agents. Overexpression of PGC-1α in C2C12 myotubes inhibits protein degradative pathways [177]. In vivo, genetic overexpression of PGC-1α in male mice mitigates some phenotypic characteristics of disuse atrophy [12], whereas local transfection of PGC-1α in the tibialis anterior of female mice mitigates some measures of oxidative stress associated with reloading after disuse [178]. Additionally, mitochondrially targeted antioxidant treatment has shown some promise in female rats for the prevention of disuse atrophies [91].

However, despite these promising findings, no current clinical treatments have yet been developed based on these findings. This may be partially due to the disparity in pre-clinical research in female models. Females generally have more mitochondria [168]; while it may be appealing to conclude that females are inherently protected against mitochondrial-related pathologies such as muscle wasting, to date this hypothesis has not been directly tested in disuse pathologies. More so, greater mitochondrial content does not necessarily translate to greater mitochondrial and cellular health, as we and others have demonstrated that genetic induction of mitochondrial biogenesis via PGC-1α overexpression does not protect against, and may exacerbate, other muscle pathologies such as insulin resistance [179–181]. Additionally, greater mitochondrial content per gram of tissue weight also presents the opportunity for greater ROS production during pathological stimuli. ROS production is thought to occur primarily through complex III within the mitochondria [182], and some evidence suggests that females may have greater complex III activity compared to males [183]. Overall suggesting that mitochondrial differences between males and females may potentially explain differences noted between males and females during disuse pathologies.

#### Mitochondria and sex hormones

It is becoming more and more accepted that the mitochondria also contain receptors specific to classical sex hormones that may influence sexual dimorphisms in mitochondrial-related pathologies. For example, estrogen receptors (specifically ERβ) have been found in the mitochondrial membrane across multiple tissues [184–190]. The ERβ is currently thought to increase transcription of nuclear- and mitochondrial-encoded proteins involved in oxidative function and of NRF-1 and COX complexes [191], leading to overall increased mitochondrial oxidation and potentially improved function [192, 193]. ERβ and ERα total content do not currently appear to differ between males and females [194, 195], thereby suggesting that ER-mediated differences between males and females are likely the result of differences in plasma hormone circulation. Androgen rectors have in some studies been noted on the mitochondria; however, this has been limited to mitochondria present in mobile sperm [196], as such, the influence ARs may have on mitochondrial and cellular function is likely limited [191]. Recently, progesterone action has been hypothesized to alter mitochondrial oxidative function. It has been established since the 1990s that during the luteal phase of the female menstrual cycle (when progesterone peaks), energy expenditure and mitochondrial respiration increase [197–199]. However, recent works have established the presence of a progesterone receptor specific to the mitochondrial membrane (PR-M) [154, 155]. This PR-M appears to facilitate increased cellular respiration within the mitochondria [154, 155], overall suggesting that progesterone may greatly influence overall mitochondrial and muscular health. However, the potential direct and indirect effects of hormonal actions and mitochondrial quality during muscle atrophies requires further investigation.

In aggregate, the current literature suggests inherent differences between males and females on both mitochondrial content and function [165, 167–170]. These differences may at least partially contribute to differential mitochondrial alterations during muscle pathologies and subsequent muscle loss [35, 46, 171]. However, many of these differences in disuse specific muscle loss have yet to be investigated as well as global differences in mitochondrial function during pathologies. As such, more research on mitochondrial-specific alterations during muscle pathologies between males and females is necessary to develop more effective therapeutics for mitochondria-related pathologies.

## Perspectives and significance

Skeletal muscle size and quality remain one of the largest mediators of overall quality of life and mortality across a variety of pathologies. Clearly, males have been preferentially researched in pre-clinical models, resulting in a relative dearth of research on muscle pathologies in females. However, the current research demonstrates that males and females are clearly different on many aspects of muscular health and physiology including muscle fiber composition, anabolic and catabolic pathways, hormonal interactions, and mitochondrial content and function. These differences can greatly influence the development and progression of various muscle atrophies, including disuse atrophy. However, specific studies investigating how any of these processes are differentially regulated between males and females in relation to muscle loss are lacking, specifically disuse studies. This lack of data has clear therapeutic implications; less than half of pharmacological agents move to phase 3 clinical trials [200], often due to lack of efficacy in human models. Part of this lack of efficacy in human models is likely partially attributable to lack of female model organisms in pre-clinical and phase I trials. If we are to fulfill the promise of individualized medicine and a more efficient and impactful health care system, we need to start seriously investigating one of the basic aspects of an individual’s genome, biological sex. Only then can we truly begin to adequately begin the long process of developing individualized medicine for patients.
